# The epidemiology of coronary artery bypass surgery in a community hospital: A comparison between 2 periods

**DOI:** 10.1097/MD.0000000000015059

**Published:** 2019-03-15

**Authors:** Tomer Ziv-Baran, Rephael Mohr, Farhang Yazdchi, Dan Loberman

**Affiliations:** a School of Public Health; b Sackler Faculty of Medicine, Tel Aviv University, Tel Aviv, Israel.; c Division of Cardiac Surgery, Brigham & Women's Hospital, Harvard Medical School, Boston, MA/Cape Cod Hospital, Hyannis, MA.

**Keywords:** coronary artery bypass surgery, comorbidities, adverse outcomes, community hospital

## Abstract

During the last decades, the increased number of percutaneous interventions procedures causes a significant change in the profile of patients referred to coronary artery bypass grafting (CABG). We aimed to study changes in patients’ characteristics and procedural outcomes of patients referred to CABG in a community hospital during the first 15 years of the millennium.

A historical cohort study of all patients who underwent CABG in Cape Cod Hospital was performed. The period was divided into 2 sub-periods, 2000 to 2008 and 2009 to 2014. Patients’ characteristics and procedure outcomes were compared. Data on age, sex, comorbidities, Society of Thoracic Surgery risk scores and surgical adverse outcomes (stroke, coma, and 30-days mortality) were collected.

During the study period, 1108 patients underwent CABG; 612 were operated before 2009 and 496 after. Age and sex were similar in the 2 periods. The patients in the later period presented lower risk for mortality and stroke (*P* <.001). Diabetes (DM) was more common in the later period (*P* <.001) while peripheral vascular disease (PVD) (*P* <.001) and left main disease (LM) (*P* = .017) were more common in the earlier period. Mortality rates were similar between the 2 periods. Post-operative stroke (1.8%) and coma (0.8%) were presented only in the later period. In conclusion, a significant change in CABG patients’ characteristics was observed.

In conclusion, patients in the later period had lower risk score and were more likely to present with DM and less with PVD and LM. Despite the lower risk, the mortality rate was similar.

## Abstract 2

Supplemental Digital Content is available in the text

## 1 Introduction

During the last decades, the epidemiology of heart surgery has changed significantly.^[1,2]^ While in its early day's percutaneous interventions (PCI) using balloon angioplasty without stents was used mainly in patients with mild disease, today most PCI procedures include multi-vessel disease and stents are the standards of care.^[3]^

Despite the fact that PCI is associated with inferior long-term outcome, it can be considered as a reasonable alternative to coronary artery bypass grafting (CABG).^[4–11]^ This revascularization technique is more popular than CABG due to its less invasive nature. The increase in number of PCI procedures causes a significant change in the profile of patients referred to CABG in recent years. The relative number of lower risk patients is increased. This is also reflected in the lower Society of Thoracic Surgery (STS) risk score of the patients who undergo surgery.^[5,12]^

The purpose of our study is to evaluate changes in patients’ characteristics and procedural outcomes between patients referred to CABG in a community hospital during the early and late periods of the first 15 years of the new millennium.

## 2 Methods

### 2.1 Study design and participants

This is a historical cohort study of all patients who underwent CABG surgery in Cape Cod Hospital (CCH) between 2000 and 2014. In our community hospital, patents are referred to CABG after being discussed by a heart team or the attending cardiologist. The indications for referral are similar to those used in other center in the USA.

The study period was divided into 2 sub-periods, 2000 to 2008 and 2009 to 2014. We compared patient's characteristics and procedure outcomes between the 2 periods.

The study was approved by the Institutional Review Board (IRB) of the CCH.

### 2.2 Setting

CCH is a 259-bed acute care community hospital located in Hyannis, Massachusetts with a 15 beds cardio-thoracic surgery department.

### 2.3 Variables and data source

Data on age, sex, comorbidities (diabetes mellitus [DM], hypertension [HTN], peripheral vascular disease [PVD], congestive heart failure [CHF], recent myocardial infarction, atrial fibrillation [A. fib] or flutter [A. flutter], left main disease [LM], and preoperative stroke), STS risk scores for mortality and stroke and surgical adverse outcomes (stroke, coma, and 30 days mortality) were obtained from review of medical records. We also used the maximal STS risk scores (mortality or stroke) and evaluated a combing outcome of mortality, stroke, or coma (MSC). Baseline patient characteristics and in-hospital outcomes were collected according to the STS Adult Cardiac Surgery Database (Data Collection Form).

### 2.4 Bias

In order to avoid selection bias, all patients who underwent surgery during the study period were included in the study. We used a standard data collection form to avoid misclassification bias.

### 2.5 Study size

A significance level of 5% and a power of 80% were used to calculate the sample size. Since the total period of the study was divided into 2 sub-periods with a ratio of 1:2, the same ratio between the groups was assumed in order to calculate the sample size that needed to identify a small difference between the groups in the continuous variables (effect size Cohen's d = 0.2) and a 10 percent difference in the dichotomous variables. Eight hundred eighty-six and 869 patients were needed to identify differences between the groups in continuous and dichotomous variables, respectively.

### 2.6 Statistical methods

Categorical variables were expressed as number and percentages. Distribution of continuous variables was assessed using histogram and Q-Q plot. Continuous variables were described using mean and standard deviation (SD) or median and interquartile range (IQR). Categorical variables were compared using Chi-square test or Fisher exact test and continuous variables using independent samples *t* test or Mann–Whitney test. Propensity score was calculated using logistic regression. Age, sex, HTN, DM, PVD, CHF, A. Fib/A. flutter, prior stroke, LM, and recent myocardial infatction were used to calculate the propensity score. Logistic regression was used to evaluate the crude and adjusted odds ratio (OR) for the MSC outcome. The multivariate logistic regression was repeated twice with different variables for adjustment (Maximal STS score, and Propensity score). In further analysis, the patients were matched according to their propensity score. Five percent difference in the propensity score was defined as maximal difference for matching. The matching process was evaluated using absolute standardized difference and difference up to 0.15 was considered as acceptable. The combined outcome was compared between the matched groups using McNemar test. The patients were also matched using their maximal STS score and 1% difference was considered as the maximal difference for matching. A 2-tailed *P* <.05 was considered statistically significant. Analyses were performed with SPSS (IBM Corp. Released 2016. IBM SPSS Statistics for Windows, Version 24.0. Armonk, NY: IBM Corp.).

## 3 Results

**Figure 1:**
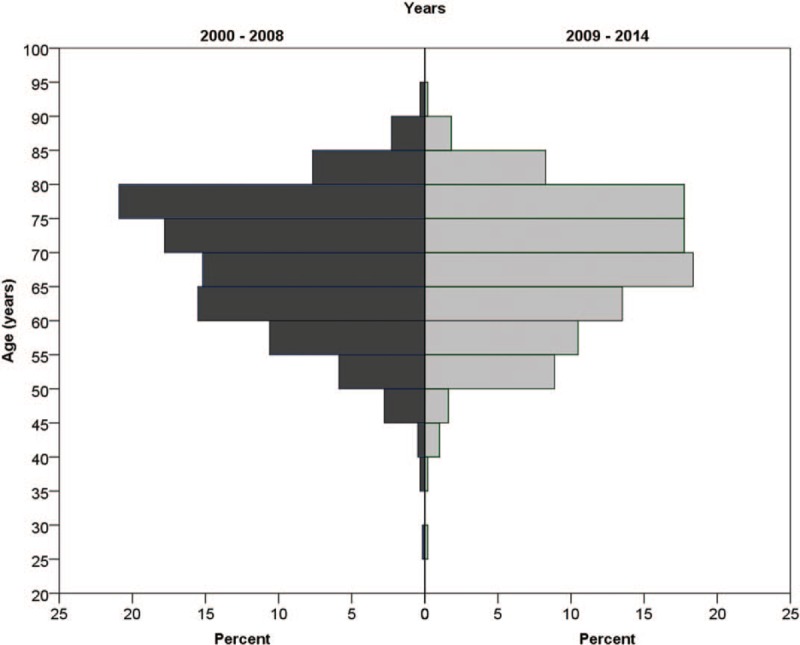
Age distribution in the 2 periods.

**Figure 2:**
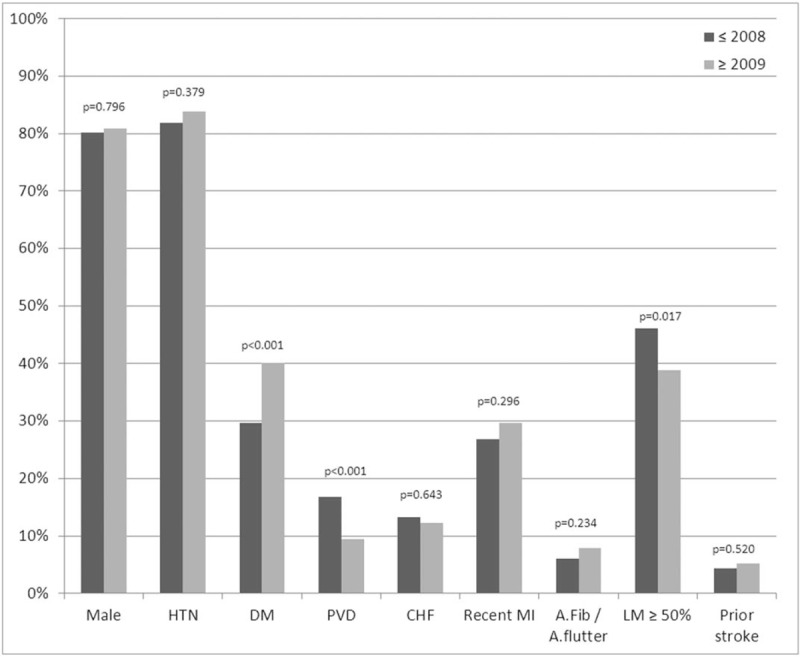
Patients’ characteristics in the 2 periods.

**Figure 3:**
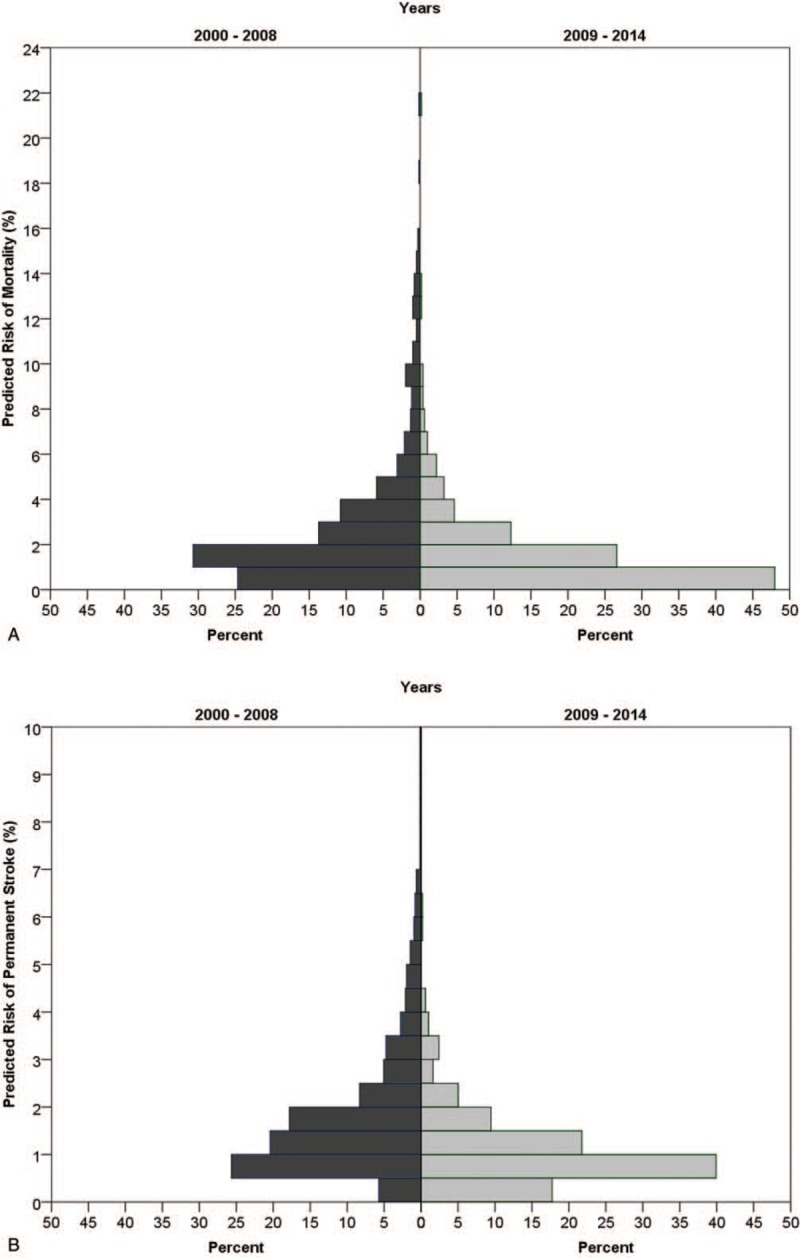
Preoperative risk for mortality (A) and stroke (B) in the 2 periods according to the STS. STS = Society of Thoracic Surgery.

The study included 1108 patients who underwent coronary artery bypass surgery. Of them, 612 were operated before 2009 and 496 after. The patients’ characteristics are presented in Table 1. Age and sex distribution were similar in the 2 periods. Patients in the later period had more DM (40.1% vs 29.7%, *P* <.001) while patients in the former period had more PVD (16.8% vs 9.5%, *P* <.001) and more LM (46.1% vs 38.9%, *P* = .017). The patients in the later period presented lower risk for mortality and lower risk for stroke as calculated by the STS score (*P* <.001). Comparison of the patients’ characteristic is presented in Table 2 and Figures 1 to 3. In the later period, post-operative strokes (1.8%), and coma (0.8%) were documented while no post-operative stroke or coma were documented in the earlier period. Mortality rates were similar between the periods (Table 3). The MSC outcome was more frequent in the later period (3.2% vs 1.1%; OR 2.88, 95% CI 1.17–7.08). After adjustment the OR (late period vs previous period) for MSC raised: maximal STS score adjustment OR 4.16 (95% CI 1.58–10.97, *P* = .004), propensity score adjustment OR 3.12 (95% CI 1.25–7.78, *P* = .014). Nine hundred ninety patients were matched (495 in each period) using the propensity score. The matched groups are described in Appendix 1. In the matched groups, the STS risk scores were significantly lower in the later period (mortality: median 1.77, IQR 0.99–3.42 vs median 1.05, IQR 0.59–2.01, *P* <.001; stroke: median 1.38, IQR 0.87–2.28 vs median 0.91, IQR 0.60–1.36, *P* <.001). The post-operative adverse outcomes of the matched groups are presented in Table 4. As in the whole cohort, post-operative stroke and coma were presented only in the later period. After matching, post-operative MSC tended to be higher in the later period (*P* = .052). In further analysis, the patients were matched using the STS score. Four hundred- seventeen matched pairs were evaluated. After this matching, there was no significant difference in the mortality (0.5% in the earlier period vs 1.7% in the later period, *P* = .125). However, the MSC outcome in the earlier period was significantly lower than that of the later period (0.5% vs 3.6, *P* = .001).

**Table 1:**
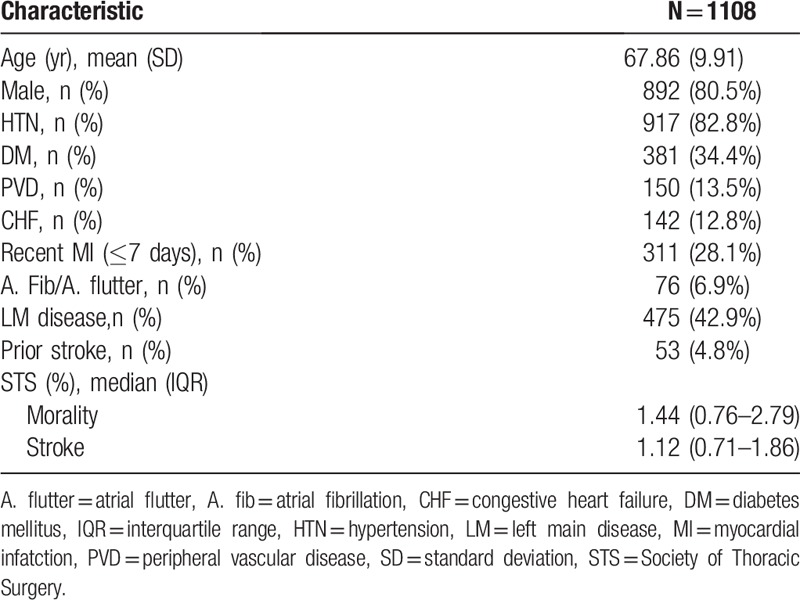
Patients’ characteristics.

**Table 2:**
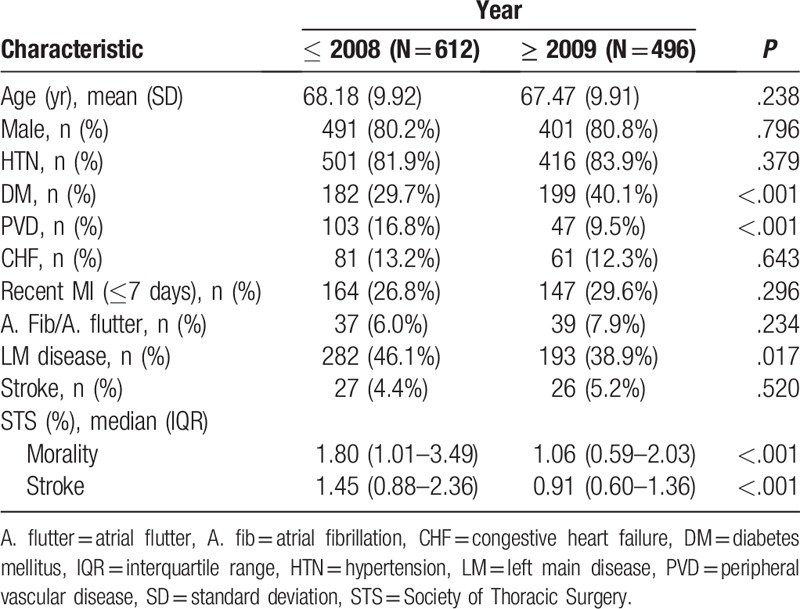
Comparison of patients’ characteristics between the 2 periods.

**Table 3:**
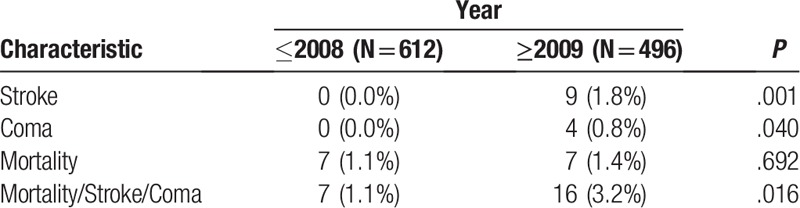
Comparison of post-operative outcomes between the 2 periods.

**Table 4:**
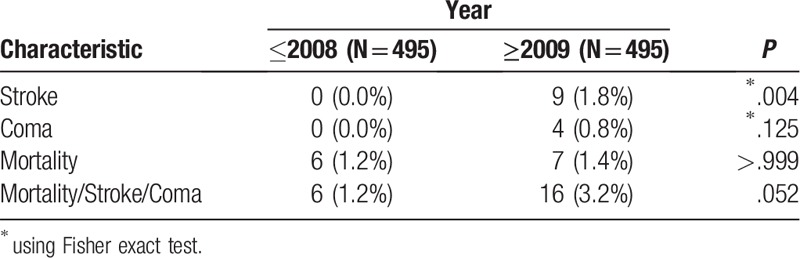
Comparison of post-operative adverse outcomes between the 2 periods in the matched cohorts.

## 4 Discussion

In this cohort study, we compared patients’ characteristics and adverse outcomes of patients operated between 2000 to 2008 and 2009 to 2014 in a community hospital.

The main finding of our study is the significant change in CABG patients’ characteristics in the later period (2009–2014). We found that DM was more common in the late period (40.1% vs 29.7%, *P* <.001) while PVD (16.8% vs 9.5%, *P* <.001) and LM disease (46.1% vs 38.9%, *P* = .017) were more common in the earlier period. The findings may be related to the global increase of DM and to better DM control or may be related to change in the profile of patients referred to CABG due to advancement in PCI procedures. Recent publication demonstrated the increased number of patients with DM during the last decade.^[13]^ Several other studies showed that better DM control is associated with a lower risk for DM complications.^[14–16]^

Patients in earlier and later periods had similar age (mean 68.2 years vs 67.5 years, *P* = .238) and sex (males: 80.2% vs 80.8%, *P* = .796). Patients in the later period were at lower risk (less PVD and LM disease) and therefore their STS Score was lower than the STS Score of patients in the earlier period. This change in CABG patients’ characteristics is probably related to advances made in PCI technology and the relatively larger number of patients refereed today to PCI. Patients referred to PCI are the higher risk patients and this selective referral policy is the reason for the relative lower risk of the patients in the later CABG group. Today, most PCI procedures include multi-vessel disease. However, in some institutions, the most severe and complex CAD patients are still referred to CABG which does not necessarily indicate the clinical complexity of the patients.

The overall number of adverse events in our cohort was low. Although the mortality rate was similar in both periods (early period 1.1%, late period 1.4%, *P* = .692), adverse neurological events were more common in the later period (stroke: 1.8% vs 0%, *P* = .001; coma: 0.8% vs 0%, *P* = .04). Previous studies reported stroke rates range between 0.8% and 5.2%^[2,17–20]^ and mortality rates between 1.5% and 6%.^[2,21–26]^ Those findings were also observed after propensity score matching. They should be evaluated further in prospective studies in order to include more possible predictors for stroke.

Our study had several limitations. Due to the historical nature of the study, we could include only data that was available in the patients’ charts (data such as echocardiographic data was not available). Moreover, we have follow-up data only for the first 30 days after surgery. Since the patients in the 2 periods were different in their pre-operative risk level and the adverse outcomes were rare, it was difficult to compare the outcomes between the 2 groups. Therefore, we used propensity score and STS score to control for differences in the baseline characteristics of the patients.

In conclusion, a significant change in CABG patients’ characteristics was observed. Patients in the later period had lower risk score and were more likely to present with DM and less with PVD and LM disease. Despite the increased operative risk of patients operated in the earlier period, their mortality is similar to that of patients operated in the later period. The higher rate of post-operative stroke reported in the later period is not different from that reported in the literature.

## Author contributions

**Conceptualization:** Rephael Mohr, Farhang Yazdchi, Dan Loberman.

**Data curation:** Tomer Ziv-Baran, Dan Loberman.

**Formal analysis:** Tomer Ziv-Baran, Farhang Yazdchi.

**Investigation:** Tomer Ziv-Baran.

**Methodology:** Tomer Ziv-Baran, Dan Loberman.

**Project administration:** Dan Loberman.

**Supervision:** Farhang Yazdchi, Dan Loberman.

**Writing – Original Draft:** Rephael Mohr.

**Writing – Review & Editing:** Farhang Yazdchi, Dan Loberman.

## Supplementary Material

Supplemental Digital Content

## References

[1] Leon MB Popma JJ Mintz GS An overview of US stent trials. Semin Interv Cardiol 1996;1:247–54. 9552518

[2] Rudersdorf PD Abolhoda A Carey JS Adverse events after coronary revascularization procedures in California 2000 to 2010. Am J Cardiol 2013;112:483–7. 23668638 10.1016/j.amjcard.2013.04.009

[3] Serruys PW Unger F van Hout BA The ARTS study (arterial revascularization therapies study). Semin Interv Cardiol 1999;4:209–19. 10738354 10.1006/siic.1999.0107

[4] Lee CW Ahn JM Cavalcante R Coronary artery bypass surgery versus drug-eluting stent implantation for left main or multivessel coronary artery disease: a meta-analysis of individual patient data. JACC Cardiovasc Interv 2016;9:2481–9. 28007199 10.1016/j.jcin.2016.10.008

[5] Kodumuri V Balasubramanian S Vij A A meta-analysis comparing percutaneous coronary intervention with drug-eluting stents versus coronary artery bypass grafting in unprotected left main disease. Am J Cardiol 2018;121:924–33. 29502793 10.1016/j.amjcard.2017.12.039

[6] Takagi H Kawai N Umemoto T Meta-analysis of four randomized controlled trials on long-term outcomes of coronary artery bypass grafting versus percutaneous coronary intervention with stenting for multivessel coronary artery disease. Am J Cardiol 2008;101:1259–62. 18435954 10.1016/j.amjcard.2007.12.026

[7] Garg A Rao SV Agrawal S Meta-analysis of randomized controlled trials of percutaneous coronary intervention with drug-eluting stents versus coronary artery bypass grafting in left main coronary artery disease. Am J Cardiol 2017;119:1942–8. 28433215 10.1016/j.amjcard.2017.03.019

[8] Sardar P Giri J Elmariah S Meta-analysis of drug-eluting stents versus coronary artery bypass grafting in unprotected left main coronary narrowing. Am J Cardiol 2017;119:1746–52. 28400029 10.1016/j.amjcard.2017.03.009

[9] Khan MR Kayani WT Ahmad W Meta-analysis of comparison of 5-year outcomes of percutaneous coronary intervention versus coronary artery bypass grafting in patients with unprotected left main coronary artery in the era of drug-eluting stents. Am J Cardiol 2017;120:1514–20. 28886851 10.1016/j.amjcard.2017.07.048

[10] Ali WE Vaidya SR Ejeh SU Meta-analysis study comparing percutaneous coronary intervention/drug eluting stent versus coronary artery bypass surgery of unprotected left main coronary artery disease: clinical outcomes during short-term versus long-term (>1 year) follow-up. Medicine (Baltimore) 2018;97:e9909–10. 29443766 10.1097/MD.0000000000009909 PMC5839846

[11] Bundhun PK Bhurtu A Chen MH Impact of coronary artery bypass surgery and percutaneous coronary intervention on mortality in patients with chronic kidney disease and on dialysis: a systematic review and meta-analysis. Medicine (Baltimore) 2016;95:e4129–30. 27399124 10.1097/MD.0000000000004129 PMC5058853

[12] Cornwell LD Omer S Rosengart T Changes over time in risk profiles of patients who undergo coronary artery bypass graft surgery: the Veterans Affairs Surgical Quality Improvement Program (VASQIP). JAMA Surg 2015;150:308–15. 25671647 10.1001/jamasurg.2014.1700

[13] Amed S Islam N Sutherland J Incidence and prevalence trends of youth-onset type 2 diabetes in a cohort of Canadian youth: 2002–2013. Pediatr Diabetes 2017;19:630–6. 29280255 10.1111/pedi.12631

[14] Nathan DM The diabetes control and complications trial/epidemiology of diabetes interventions and complications study at 30 years: overview. Diabetes Care 2014;37:9–16. 24356592 10.2337/dc13-2112 PMC3867999

[15] Dailey G Early and intensive therapy for management of hyperglycemia and cardiovascular risk factors in patients with type 2 diabetes. Clin Ther 2011;33:665–78. 21704233 10.1016/j.clinthera.2011.04.025

[16] Nathan DM Genuth S Lachin J The effect of intensive treatment of diabetes on the development and progression of long-term complications in insulin-dependent diabetes mellitus. N Engl J Med 1993;329:977–86. 8366922 10.1056/NEJM199309303291401

[17] Lee EJ Choi KH Ryu JS Stroke risk after coronary artery bypass graft surgery and extent of cerebral artery atherosclerosis. J Am Coll Cardiol 2011;57:1811–8. 21527154 10.1016/j.jacc.2010.12.026

[18] Tarakji KG Sabik JF 3rd Bhudia SK Temporal onset, risk factors, and outcomes associated with stroke after coronary artery bypass grafting. JAMA 2011;305:381–90. 21266685 10.1001/jama.2011.37

[19] Mehta A Choxi R Gleason T Carotid artery disease as a predictor of in-hospital postoperative stroke after coronary artery bypass grafting from 1999 to 2011. J Cardiothorac Vasc Anesth 2017 2011;S1053-0770:30794–802. 10.1053/j.jvca.2017.10.010 29169797

[20] Moreyra AE Maniatis GA Gu H Frequency of stroke after percutaneous coronary intervention or coronary artery bypass grafting (from an eleven-year statewide analysis). Am J Cardiol 2017;119:197–202. 27817795 10.1016/j.amjcard.2016.09.046

[21] Hansen LS Hjortdal VE Andreasen JJ 30-day mortality after coronary artery bypass grafting and valve surgery has greatly improved over the last decade, but the 1-year mortality remains constant. Ann Card Anaesth 2015;18:138–42. 25849679 10.4103/0971-9784.154462 PMC4881647

[22] Hannan EL1 Racz MJ Walford G Long-term outcomes of coronary-artery bypass grafting versus stent implantation. N Engl J Med 2005;352:2174–83. 15917382 10.1056/NEJMoa040316

[23] Birkmeyer JD Siewers AE Finlayson EV Hospital volume and surgical mortality in the United States. N Engl J Med 2002;346:1128–37. 11948273 10.1056/NEJMsa012337

[24] Peterson ED Coombs LP DeLong ER Procedural volume as a marker of quality for CABG surgery. JAMA 2004;291:195–201. 14722145 10.1001/jama.291.2.195

[25] Nallamothu BK Saint S Hofer TP Impact of patient risk on the hospital volume-outcome relationship in coronary artery bypass grafting. Arch Intern Med 2005;165:333–4. 15710799 10.1001/archinte.165.3.333

[26] Cram P Rosenthal GE Vaughan-Sarrazin MS Cardiac revascularization in specialty and general hospitals. N Engl J Med 2005;352:1454–62. 15814881 10.1056/NEJMsa042325

