# The epidemiology of coronary artery bypass surgery in a community hospital: A comparison between 2 periods: Erratum

**DOI:** 10.1097/MD.0000000000015455

**Published:** 2019-04-26

In the article, “The epidemiology of coronary artery bypass surgery in a community hospital: A comparison between 2 periods”^[1]^, which appears in Volume 98, Issue 13 of *Medicine*, affiliation a appears incorrectly and should be “Deptartment of Epidemiology and Preventive Medicine, School of Public Health, Sackler Faculty of Medicine, Tel Aviv University, Tel Aviv, Israel.”
