# Supplementary material for: The epidemiology of coronary artery bypass surgery in a community hospital: A comparison between 2 periods
Source: Medicine (Baltimore). 2019 Mar 15;98(13):e15059. doi: 10.1097/MD.0000000000015059 (PMC6455745; doi:10.1097/MD.0000000000015059)
Supplement: Supplemental Digital Content [file medi-98-e15059-s001.docx]

**Appendix:**

Comparison of patients’ characteristics after matching

| **Characteristic** | **Year** | | **Absolute Standardized Difference** |
| --- | --- | --- | --- |
|  | **≤ 2008** | **≥ 2009** |  |
|  | **(N=495)** | **(N=495)** |  |
| Age (years), mean (SD) | 67.68 (10.02) | 67.44 (9.90) | 0.0241 |
| Male, n(%) | 399 (80.6%) | 400 (80.8%) | 0.0051 |
| HTN, n(%) | 407 (82.2%) | 416 (84.0%) | 0.0486 |
| DM, n(%) | 165 (33.3%) | 198 (40.0%) | 0.1387 |
| PVD, n(%) | 61 (12.3%) | 47 (9.5%) | 0.0908 |
| CHF, n(%) | 65 (13.1%) | 61 (12.3%) | 0.0242 |
| Recent MI (≤7 days), n(%) | 140 (49.0%) | 146 (29.5%) | 0.0267 |
| A.Fib / A.flutter, n(%) | 31 (44.9%) | 38 (55.1%) | 0.0556 |
| LM disease, n(%) | 218 (44.0%) | 193 (39.0%) | 0.1026 |
| Prior stroke, n(%) | 21 (4.2%) | 25 (5.1%) | 0.0384 |
